# *Lactobacillus acidophilus* LA14 Alleviates Liver Injury

**DOI:** 10.1128/mSystems.00384-21

**Published:** 2021-06-15

**Authors:** Longxian Lv, Chunyan Yao, Ren Yan, Huiyong Jiang, Qiangqiang Wang, Kaicen Wang, Siqi Ren, Shandong Jiang, Jiafeng Xia, Shengjie Li, Ying Yu

**Affiliations:** a State Key Laboratory for Diagnosis and Treatment of Infectious Diseases, Collaborative Innovation Center for Diagnosis and Treatment of Infectious Diseases, The First Affiliated Hospital, College of Medicine, Zhejiang University, Hangzhou, China; b Key Laboratory of Nutrition of Zhejiang Province, Institute of Health Food, Hangzhou Medical College, Hangzhou, China; c School of Pharmacy, Hangzhou Medical College, Hangzhou, China; d Department of Clinical Medicine, Hangzhou Medical College, Hangzhou, China; Drexel University

**Keywords:** acute liver injury, *Lactobacillus acidophilus*, microbiota, metabolome, transcriptome

## Abstract

Although the probiotic Lactobacillus acidophilus LA14 is used worldwide, its effect on liver diseases remains unelucidated. Here, 32 rats were divided into four groups, gavaged with L. acidophilus LA14 (3 × 10^9^ CFU) or phosphate-buffered saline for 7 days, and then intraperitoneally injected with d-galactosamine or saline. After 24 h, blood, liver, ileum, and feces samples were collected for liver injury, inflammation, intestinal barrier, gut microbiota, metabolome, and transcriptome analyses. Pretreatment with L. acidophilus LA14 alleviated the d-galactosamine-induced elevation of serum alanine aminotransferase (ALT), aspartate aminotransferase (AST), alkaline phosphatase (ALP), and bile acids; mitigated the histological injury to the liver and gut; and suppressed the inflammatory cytokines macrophage inflammatory protein 1α (MIP-1α), MIP-3α, and MCP-1. L. acidophilus LA14 also ameliorated the d-galactosamine-induced dysbiosis of the gut microbiota and metabolism, such as the enrichment of *Bacteroides* sp. strain dnLKV3 and the depletion of Streptococcus, butanoic acid, and *N-*acetyl-d-glucosamine. The underlying mechanism of L. acidophilus LA14 included prevention of not only the d-galactosamine-induced upregulation of infection- and tumor-related pathways but also the d-galactosamine-induced downregulation of antioxidation-related pathways during this process, as reflected by the liver transcriptome and proteome analyses. Furthermore, the administration of L. acidophilus LA14 to healthy rats did not alter the tested liver indicators but significantly enriched the beneficial *Lactobacillus* and *Bifidobacterium* species, promoted metabolism and regulated pathways to improve immunity. The ability of L. acidophilus LA14 to alleviate liver injury was further confirmed with an acetaminophen-induced mouse model. These results might provide a reference for future studies on the application of L. acidophilus LA14 for the prevention of liver injury.

**IMPORTANCE** The probiotic Lactobacillus acidophilus LA14 is widely used, but its effect on liver diseases has not been elucidated. We explored the protective effect of L. acidophilus LA14 on the liver using rats with d-galactosamine-induced liver injury. Pretreatment with L. acidophilus LA14 alleviated the d-galactosamine-induced elevation of serum ALT, AST, ALP, and bile acids, mitigated the histological injury to the liver and gut, and suppressed the inflammatory cytokines MIP-1α, MIP-3α, and MCP-1. These effects were correlated with the modulations of the gut microbiome, metabolome, and hepatic gene expression induced by L. acidophilus LA14. Moreover, the ability of L. acidophilus LA14 to alleviate liver injury was further confirmed with an acetaminophen-induced mouse model. These results might provide a reference for future studies on the application of L. acidophilus LA14 for the prevention of liver injury.

## INTRODUCTION

Acute liver injury is a frequently observed clinical syndrome. In the absence of intervention, this injury will develop into fatal acute liver failure, which has a high mortality of 60 to 80% and a yearly incidence of one to six cases per million people worldwide (1). The human gut microbiota is composed of more than 1,000 bacterial species with a high concentration of up to 10^12^ cells per g of feces (2). Accumulating evidence shows strong links between alterations in the gut microbiota and the process of liver injury (3). Alterations in liver functions, such as bile secretion and gastrointestinal peristalsis, disturb the gut microbiota (2, 4). Gut microbiota dysbiosis is associated with decreases in beneficial bacteria, such as *Lactobacillus* and *Bifidobacterium* spp., and potential increases in opportunistic pathogens, such as *Enterococcus*, and their products, which will aggravate liver diseases (3). Thus, therapies to modulate the gut microbiota and their products are necessary for the prevention and treatment of acute liver injury.

Several probiotics show good performance in the treatment of liver diseases. For instance, Lactobacillus rhamnosus LGG ameliorates liver injury and hypoxic hepatitis in a rat model of cecal ligation and puncture (CLP)-induced sepsis (5), and Lactobacillus plantarum CMU995 ameliorates alcohol-induced liver injury by improving both the intestinal barrier and antioxidant activity (6). Our previous studies revealed that *L. salivarius* strain LI01, Pediococcus pentosaceus LI05, and Bifidobacterium adolescentis CGMCC 15058 can alleviate hepatic injury by modulating the gut microbiota and/or their metabolites (7–9). In contrast, some lactic acid bacteria reportedly aggravate liver injury induced by d-galactosamine (d-GalN) in Sprague-Dawley rats (8, 10). Therefore, it is important to fully elucidate the effects of widely used probiotics on liver diseases.

Lactobacillus acidophilus LA14 (Danisco) is a widely used commercial probiotic that can produce bacteriocin against L. monocytogenes *in vitro* (11) and attenuate Gardnerella vaginalis-induced bacterial vaginosis by regulating both vaginal and systemic innate and adaptive immune responses *in vivo* (12). However, the effect of this strain on liver diseases has not been determined. Here, the role and mechanism of Lactobacillus acidophilus LA14 in acute liver injury were evaluated using a d-galactosamine-injected rat model and were then validated using an acetaminophen-gavaged mouse model.

## RESULTS

### *L. acidophilus* LA14 attenuates d-GalN-induced liver injury.

**FIG 1:**
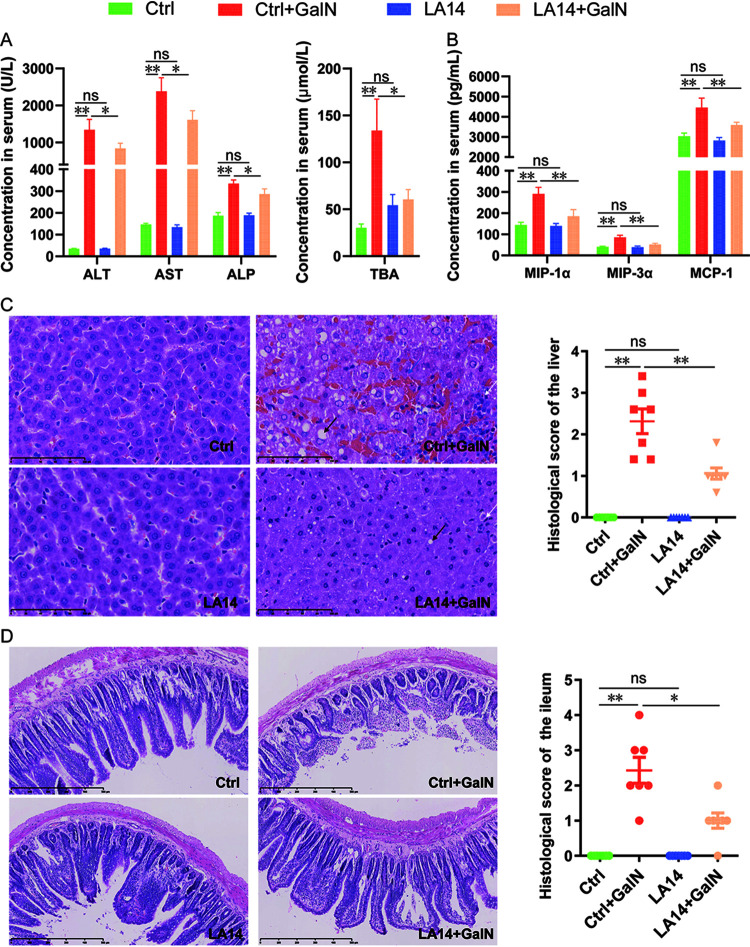
L. acidophilus LA14 attenuated d-galactosamine-induced liver and ileum injury. (A) Liver function of each group. (B) Hepatic inflammatory cytokines MIP-1α, MIP-3α, and MCP-1. (C) Representative images of hepatic HE staining and histological scores of the livers. Scale bar, 100 μm. The black arrow indicates a lipid vacuole, the blue arrow indicates hemorrhage, and the white arrow points to neutrophil and macrophage accumulation. (D) L. acidophilus LA14 alleviated d-galactosamine-induced terminal ileum injury. Representative images of ileal HE staining and histological scores of the ileums. Scale bar, 100 μm. The data are shown as the means ± the SEM (*, *P* < 0.05; **, *P* < 0.01; ns, the difference is not significant).

Twenty-four hours after the induction of liver injury, no mortality was observed in the different experimental groups. The injection of d-GalN decreased the serum levels of total protein, albumin and globulin but increased the serum levels of alanine aminotransferase (ALT), aspartate aminotransferase (AST), alkaline phosphatase (ALP), and total bile acids (TBAs) (Fig. 1A; see also Fig. S1 in the supplemental material). Pretreatment with L. acidophilus LA14 significantly lowered the d-GalN-induced increase in serum ALT, AST, ALP, and TBAs (Fig. 1A) but did not cause any significant changes in our tested liver functions in the healthy control (Ctrl) group, which indicated that LA14 safely attenuated d-GalN-induced liver injury.

10.1128/mSystems.00384-21.1 FIG S1 Liver function indicators altered by d-GalN but not alleviated by LA14 in rats. Download FIG S1, TIF file, 6.7 MB. Copyright © 2021 Lv et al. 2021 Lv et al. https://creativecommons.org/licenses/by/4.0/ This content is distributed under the terms of the Creative Commons Attribution 4.0 International license.

The effects of L. acidophilus LA14 on d-GalN-induced liver histological abnormalities were examined by hematoxylin and eosin (HE) staining. Rats injected with d-GalN exhibited hepatic histological abnormalities, such as lipid vacuole accumulation, hemorrhage, and extensive neutrophil and macrophage accumulation in the parenchyma (Fig. 1C). The administration of LA14 alone did not induce histological abnormalities in the healthy rats but reduced d-GalN-induced liver damage (Fig. 1C), which further indicated that LA14 reduced d-GalN-induced liver injury.

### *L. acidophilus* LA14 reduces the d-GalN-induced increase in macrophage inflammatory proteins and macrophage chemotactic protein in serum.

Among the 23 inflammatory cytokines tested, significant increases in the serum levels of interleukin-1α (IL-1α), IL-1β, IL-2, IL-4, IL-5, IL-6, IL-7, IL-10, IL-12, IL-13, IL-17, IL-18, granulocyte colony-stimulating factor (G-CSF), gamma interferon (IFN-γ), regulated upon activation normal T-cell expressed and secreted (RANTES), tumor necrosis factor alpha (TNF-α), macrophage inflammatory proteins-1α (MIP-1α) and MIP-3α, and monocyte chemoattractant protein 1 (MCP-1), and decreased levels of growth-related oncogene (GRO/KC) were detected 24 h after d-GalN injection. Pretreatment with L. acidophilus LA14 did not change the levels of any of the tested cytokines in healthy rats but alleviated the d-GalN-induced increases in MIP-1α, MIP-3α, and MCP-1 (Fig. 1B). These results indicated the potential involvement of monocytes/macrophages in the LA14-mediated attenuation of d-GalN-induced liver injury.

### *L. acidophilus* LA14 alleviates d-GalN-induced terminal ileum injury.

The integrity of the intestinal mucosa plays an important role in the maintenance of intestinal mucosal barrier function, which prevents gut microbes and their toxins from migrating to extraintestinal tissues and organs (13). A high incidence of subepithelial Gruenhagen’s space and denuded villi were observed in the Ctrl+GalN group, which indicated that the intestinal mucosa integrity was damaged. Denuded villi had disappeared in the LA14+GalN group, and the incidence of subepithelial Gruenhagen’s space was decreased in this group (Fig. 1D) compared to that in the Ctrl+GalN group. This finding indicated that LA14 significantly reduced d-GalN-induced terminal ileum injury.

### *L. acidophilus* LA14 ameliorates d-GalN-induced microbiota dysbiosis.

**FIG 2:**
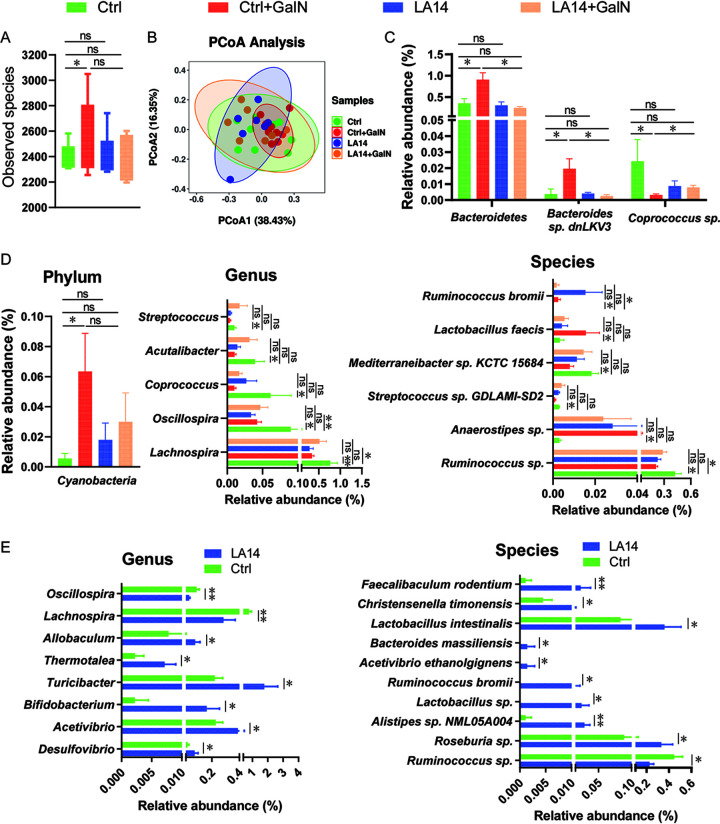
L. acidophilus LA14 alleviated d-galactosamine-induced gut microbiota dysbiosis. (A) Differences in the amounts of observed species. The values are expressed as the medians with interquartile ranges. (B) PCoA plot with weighted UniFrac distance based on OTUs of the gut microbiota. (C) The relative abundances of gut bacteria were altered after d-GalN injection but significantly reversed by L. acidophilus LA14. (E) The relative abundance of gut bacterial taxa differed between the Ctrl and Ctrl+GalN groups but not between the Ctrl+GalN and LA14+GalN groups. (F) The relative abundance of genera and species in healthy rats was significantly altered after the oral administration of L. acidophilus LA14. The data are shown as means ± the SEM (*, *P* < 0.05; **, *P* < 0.01; ns, the difference is not significant).

To explore whether the alleviation of liver injury by LA14 is linked to the regulation of gut microbiota, next-generation sequencing of the 16S rRNA V3-V4 regions of the fecal microbiota was conducted. A total of 1,891,733 reads from 32 samples of rat feces were filtered for downstream analysis. Based on ≥97% sequence identity, 5,883 qualified operational taxonomic units (OTUs) were clustered. The analysis of α-diversity, which indicates the community richness, revealed that LA14 prevented the increase in observed species induced by d-GalN (Fig. 2A), and the assessment of β-diversity, which examines the structural differences in the microbiota community, revealed significant Bray-Curtis principal coordinate analysis (PCoA) results (*P* = 0.004) (Fig. 2B), which indicated significant differences in the microbiota compositions among the four groups.

Pretreatment with LA14 significantly ameliorated some d-GalN-induced alterations in the gut microbiota. At the species level, the injection of d-GalN induced enrichment of *Bacteroidetes* sp. and *Bacteroides* sp. strains dnLKV3 and depletion of *Coprococcus* sp. Notably, these enrichments or depletions were significantly prevented by the oral administration of LA14 prior to the injection of d-GalN (Fig. 2C). Furthermore, no significant differences in the distribution of the above-mentioned bacterial taxa were found between the Ctrl and LA14 groups, which indicated that LA14 had no significant influence on these bacteria in healthy rats.

Pretreatment with LA14 made some of the d-GalN-induced alterations in the gut microbiota not significant. d-GalN induced the depletion of *Lachnospira*, *Oscillospira*, *Coprococcus*, *Acutalibacter*, Streptococcus, *Mediterraneibacter* sp. strain KCTC 15684, Streptococcus sp. strain GDLAMI-SD2, and *Ruminococcus* sp. and the enrichment of *Cyanobacteria*, Ruminococcus bromii, Lactobacillus faecis, and *Anaerostipes* sp. The relative abundances of these taxa were not significantly different between the Ctrl+GalN and LA14+GalN groups, which indirectly indicated that pretreatment with LA14 alleviated the d-GalN-induced alterations in these gut bacteria.

In healthy rats, LA14 did not affect the distribution of most of the above-mentioned bacteria. Specifically, LA14 depleted fecal *Lachnospira*, *Oscillospira*, and Ruminococcus bromii but enriched *Allobaculum*, *Acetivibrio*, *Bifidobacterium*, *Desulfovibrio*, *Thermotalea*, *Turicibacter*, Acetivibrio ethanolgignens, *Alistipes* sp. strain NML05A004, Bacteroides massiliensis, *Christensenella timonensis*, Faecalibaculum rodentium, Lactobacillus intestinalis, *Lactobacillus* sp., *Ruminococcus* sp., and *Roseburia* sp. (Fig. 2D and E).

### *L. acidophilus* LA14 relieves d-GalN-induced fecal metabolome dysbiosis.

**FIG 3:**
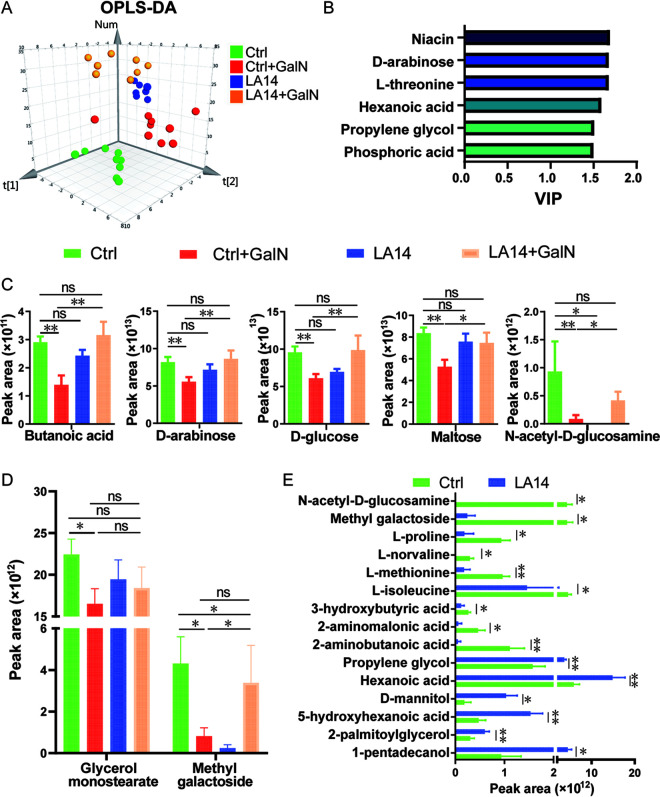
L. acidophilus LA14 alleviated d-galactosamine-induced gut metabolome dysbiosis. (A) OPLS-DA score plots of metabolome profiles of the four groups. (B) VIP values with jack-knifed confidence intervals. Only metabolites with a VIP of >1.5 are shown. (C) Peak areas of metabolites altered after d-GalN injection but significantly reversed by L. acidophilus LA14. (D) Peak areas of metabolites different between the Ctrl and Ctrl+GalN groups but not between the Ctrl+GalN and LA14+GalN groups. (E) Peak areas of metabolites significantly altered after the oral administration of L. acidophilus LA14 in healthy rats. The data are shown as means ± the SEM (*, *P* < 0.05; **, *P* < 0.01; ns, the difference is not significant).

To explore the relationship between alterations in gut metabolites and liver injury, a metabolome analysis of 32 fecal samples was performed using GC-MS. In total, 118 metabolites were identified. An orthogonal projections to latent structures and classification analysis (OPLS-DA) showed that the metabolome profiles of each group were clearly separated from those of the other three groups (Fig. 3A), which indicated that the metabolome compositions of these four groups were different. The use of the variable importance in the projection (VIP) score >1.5 as the threshold revealed that niacin, d-arabinose, l-threonine, hexanoic acid, propylene glycol, and phosphoric acid strongly contributed to the discrimination of these four groups (Fig. 3B).

Pretreatment with LA14 significantly reversed some d-GalN-induced fecal metabolic alterations. Five fecal metabolites, namely, butanoic acid, d-arabinose, d-glucose, maltose, and *N-*acetyl-d-glucosamine, were depleted in the d-GalN group compared to the control group, but their distributions did not significantly differ between the Ctrl+GalN and LA14+GalN groups. Furthermore, the oral administration of LA14 alone did not cause alterations in the five above-mentioned metabolites in healthy rats. These results suggested that the maintenance of intestinal metabolic homeostasis might be involved in the mechanisms through which LA14 alleviates d-GalN-induced liver injury.

Pretreatment with LA14 also made some of the d-GalN-induced alterations in fecal metabolites nonsignificant. For example, d-GalN induced the depletion of gut glycerol monostearate and methyl galactoside, but the distributions of these two compounds did not differ between the Ctrl+GalN and LA14+GalN groups or between the Ctrl and LA14 groups (Fig. 3D). These results further indicated that the prevention of gut metabolism dysbiosis was associated with the mechanism of LA14-alleviated liver injury.

In healthy rats, LA14 increased the propylene glycol, hexanoic acid, d-mannitol, 5-hydroxyhexanoic acid, 2-palmitoylglycerol, and 1-pentadecanol levels but decreased the levels of *n*-acetylglucosamine, methyl galactoside, l-proline, l-norvaline, l-methionine, l-isoleucine, 3-hydroxybutyric acid, 2-aminomalonic acid, and 2-aminobutanoic acid in feces (Fig. 3E). These LA14-induced alterations in the metabolome might contribute to the ability of the probiotic to alleviate d-GalN-induced liver injury.

### *L. acidophilus* LA14 alleviates d-GalN-induced upregulation of hepatic transcripts in tumor-related pathways and downregulation of hepatic transcripts in antioxidation-related pathways.

**FIG 4:**
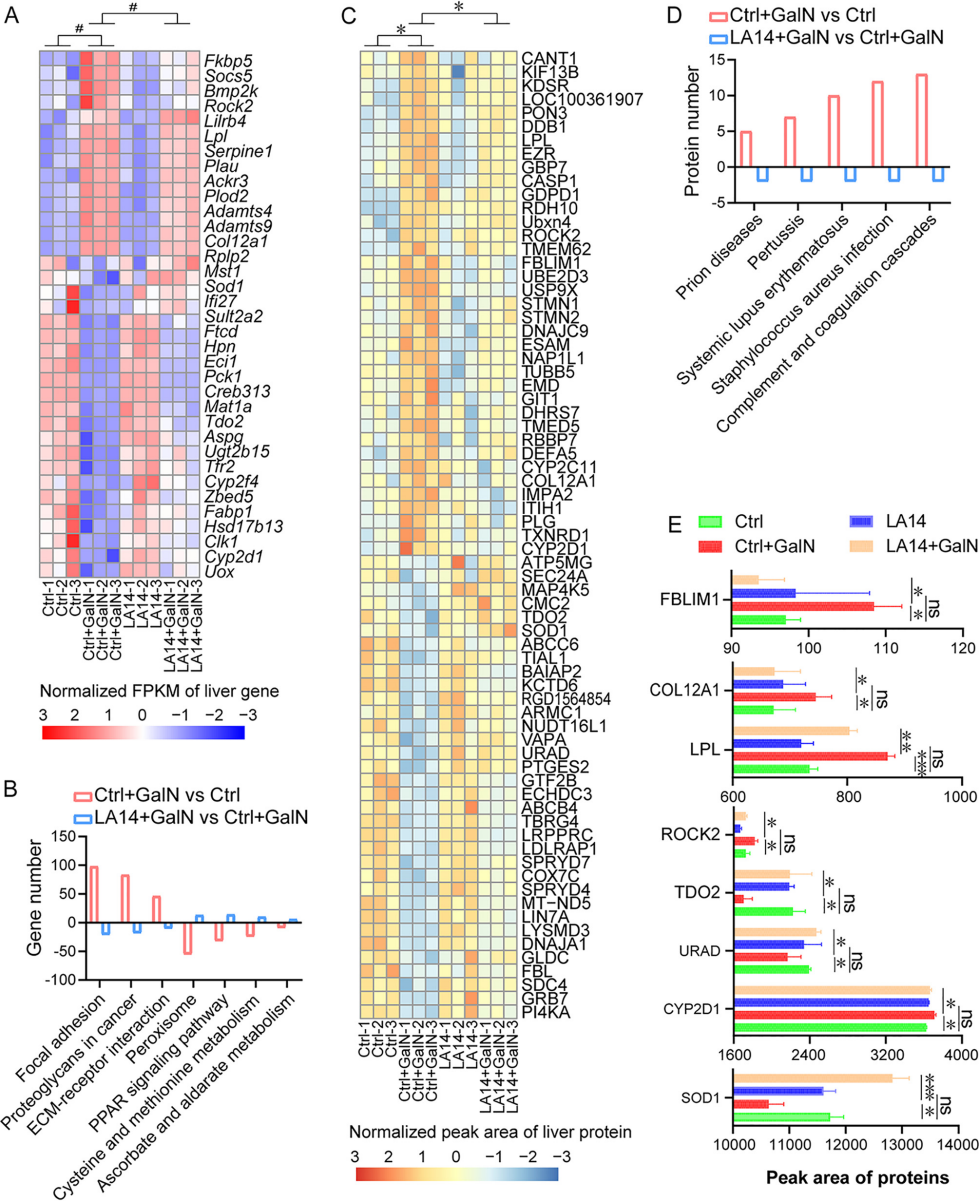
L. acidophilus LA14 regulated the transcription and/or expression of certain genes in the liver. (A and B) Genes (A) and KEGG pathways (B) that were altered after d-GalN injection but significantly alleviated by LA14 at the transcriptional level. FPKM, fragments per kilobase per million; #, *P*_adj_ < 0.05. (C and D) Proteins (C) and their related KEGG pathways (D) that were altered after d-GalN injection but significantly alleviated by LA14. *, *P* < 0.05. (E) Proteins of eight genes altered by d-GalN but alleviated by LA14 at both the transcription and expression levels. The data are shown as means ± the SEM (*, *P* < 0.05; **, *P* < 0.01; ns, the difference is not significant).

Genome-wide transcriptional profiling of the liver and gut was performed to explore which genes contributed to the LA14-mediated alleviation of liver injury. Using the absolute value of log_2_(fold change) > 1 and an adjusted *P* value (*P*_adj_) < 0.05 as the criteria, we found that the transcripts of 35 hepatic genes were altered by d-GalN but alleviated by LA14 (Fig. 4A). First, pretreatment with LA14 alleviated the d-GalN-induced upregulation of the transcription of 13 genes in the liver, including biomarkers of cytokine secretion (*Lilrb4* and *Ackr3*), cell carcinoma growth (*Rock2*, *Socs5*, *Serpine1*, *Plau*, *Plod2*, and *Col12a1*), cardiovascular disease (*Adamts4* and *Adamts9*), stress (*Fkbp5*), bone development (*Bmp2k*), and lipid metabolism (*Lpl*) (Fig. 4A). Second, pretreatment with LA14 also relieved the d-GalN-induced downregulation of the transcription of 22 genes: most of these genes are involved in immunity regulation (*MstI*, *Ifi27*, and *Aspg*), cancer suppression (*Ftcd*, *Hpn*, *Pck1*, *Mat1a*, *Tdo2*, and *Clk1*), and lipid metabolism (*Eci1*, *Creb3l3*, *Fabp1*, and *Hsd17b13*), and some of the genes are relevant to reduced secretion of hepatic bile acid (*Cyp2f4* and *Cyp2d1*), increased detoxification (*Sult2a2* and *Ugt2b15*), iron homeostasis (*Tfr2*), antiaging (*Rplp2*), antioxidant activity (*Sod1*), and urinary excretion (*Uox*).

A Kyoto Encyclopedia of Genes and Genomes (KEGG) pathway analysis of the liver revealed that d-GalN induced the upregulation of 64 pathways and the downregulation of 25 pathways (Fig. 4B, *P*_adj_ < 0.05). Pretreatment with LA14 alleviated the upregulation of the focal adhesion pathway, the ECM (extracellular matrix)-receptor interaction pathway and the proteoglycans in the cancer pathway as well as the downregulation of the ascorbate and aldarate metabolism pathway, the cysteine and methionine metabolism pathway, the PPAR signaling pathway, and the peroxisome pathway.

Using the above-mentioned screening threshold, we found that pretreatment with LA14 only alleviated the d-GalN-induced transcript upregulation of *Tma7* in the gut. However, the transcript-level analysis did not reveal an intestinal pathway that was significantly altered by d-GalN and alleviated by LA14.

In healthy rats, the administration of LA14 did not alter gene transcription in the liver (*P*_adj_ < 0.05), but this treatment altered the transcription of 1,737 genes in the intestine (*P*_adj_ < 0.05), and the top 30 genes ranked by fold change in transcription are shown in Fig. S2A. Most of the 30 genes were upregulated and involved in beneficial immunity regulation, such as reducing the risk of tumorigenesis (*Acida*, *Pox1*, *Pox5*, *Il24*, and *Siglec10*), anti-inflammation (*Ccl19* and *Acod1*), resistance to microbial infection (*Ifit1* and *Defb52*), differentiation of T and B cells (*Ms4a1* and *Cd79al*), and antigen presentation (*Rt1-Db2*). Moreover, the most differentially transcribed gene, *Mptx*, has been postulated to be involved in the processing of dying cells and thereby in reducing colon cancer risk (14). In addition, the *Klrel* gene, which was downregulated by LA14, has immunosuppressive properties of inhibiting natural killer cells. The KEGG analysis showed that 31 intestinal pathways were significantly regulated by LA14 in healthy rats (*P*_adj_ < 0.05) (see Fig. S2B). Most of these pathways were found to be related to immune regulation, including resistance to cancer, prevention of infection, and maintenance of the immune system, and some were related to tissue repair and ion balance. These results suggested that LA14 could improve the immunity of healthy rats.

10.1128/mSystems.00384-21.2 FIG S2 (A) Top 30 gut genes with the highest fold change in transcription after the oral administration of L. acidophilus LA14 in healthy rats. (B) KEGG pathways altered by L. acidophilus LA14 in the gut of healthy rats. FPKM, fragments per kilobase per million; #, *P*_adj_ < 0.05; ##, *P*_adj_ < 0.01. Download FIG S2, TIF file, 13.5 MB. Copyright © 2021 Lv et al. 2021 Lv et al. https://creativecommons.org/licenses/by/4.0/ This content is distributed under the terms of the Creative Commons Attribution 4.0 International license.

### *L. acidophilus* LA14 alleviates d-GalN-induced hepatic expression upregulation in pathways related to infections or systemic lupus erythematosus.

A proteomic analysis was performed to further explore the potential mechanism through which LA14 alleviated d-GalN-induced acute liver injury. In general, unadjusted *P* values were used to select significantly differentially expressed proteins because gene expression showed lower changes compared to transcription. The levels of 71 proteins were not only altered by d-GalN injection but also became nonsignificantly altered at the *P* < 0.05 level after pretreatment with LA14. First, LA14 alleviated the d-GalN-induced upregulation of 37 hepatic proteins (Fig. 4C), which included tumor-associated biomarkers (TXNRD1, GIT1, TMED5, IMPA2, RBBP7, EMD, NAP1L1, STMN1, ITIH1, FBLIM1, USP9X, EZR, CANT1, STMN2, ROCK2, and COL12A1), immune suppressors (KIF13B, TUBB5, UBE2D3, GBP7, ESAM, PLG, and CASP1), inducers of hepatic injury (KDSR and DEFA5), lipid metabolic proteins (PON3, DHRS7, TMEM62, DDB1, RDH10, LPL, and CYP2D1), antioxidant molecules (CYP2C11 and DNAJC9), and proteins with unknown function (UBXN4, GDPD1, and LOC100361907). Second, LA14 also relieved the d-GalN-induced downregulation of 34 hepatic proteins, which consisted of tumorigenesis factors (GTF2B, SDC4, TBRG4, COX7C, FBL, TOD2, LIN7A, MAP4K5, GRB7, SPRYD4, VAPA, and SEC24A), immunosuppression factors (DNAJA1, GLDC, CMC2, and TIAL1), energy-related factors (LRPPRC, ABCB4, MT-ND5, ABCC6, ATP5MG, and ARMC1), and proteins related to dyslipidemia (LDLRAP1, ECHDC3, PTGES2, and PI4KA), oxidative stress (SOD1), ion channel dysfunction (KCTD6), and other unknown physiological reactions (SPRYD7, BAIAP2, LYSMD3, and RGD1564854).

A KEGG analysis at the protein level revealed that the injection of d-GalN upregulated 59 pathways and downregulated 25 pathways in the liver (Fig. 4D, *P*_adj_ < 0.05). LA14 alleviated the d-GalN-induced upregulation of complement and coagulation cascades, prion diseases, pertussis, Staphylococcus aureus infection and systemic lupus erythematosus but did not alleviate any downregulated pathway.

In healthy rats, the oral administration of L. acidophilus LA14 completely altered the expression of 793 hepatic genes at the *P* < 0.05 level, but no pathways were significantly altered at the *P*_adj_ < 0.05 level.

The comparative analysis of the proteome and transcriptome revealed that eight genes (*Rock2*, *Col12a1*, *Lpl*, *Tdo2*, *Cyp2d1*, *Sod1*, *Urad*, and *Fblim1*) were altered by d-GalN and alleviated by LA14 at both the transcriptional and expression levels (Fig. 4E). The proteins encoded by these genes were mainly involved in pathways related to tumor progression, lipid disorder, and antioxidant activity (15).

### Correlations among the gut microbiota, gut metabolome, gene expression in the liver, and liver injury in the rat model.

**FIG 5:**
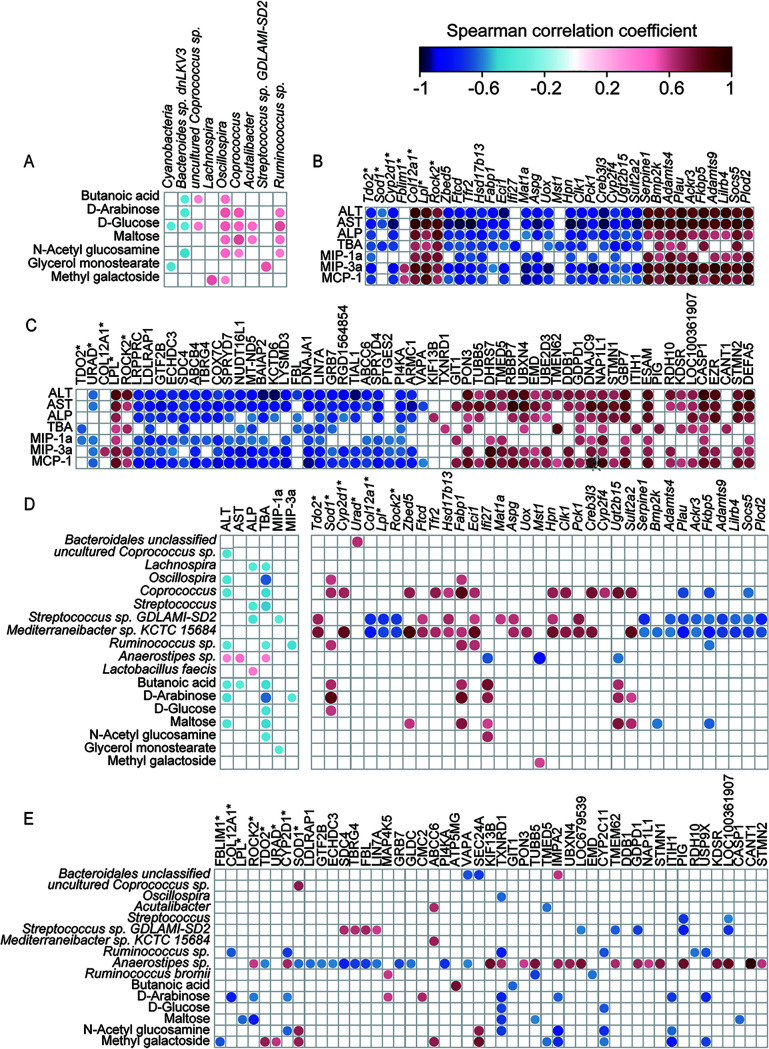
(A) Correlations of altered gut bacteria with altered gut metabolites. (B) Correlations of altered liver function indicators and inflammatory cytokines with hepatic genes altered at the transcription level. (C) Correlations of altered liver function indicators and inflammatory cytokines with altered hepatic proteins. (D) Correlations of altered gut bacteria and gut metabolites with altered liver function indicators, inflammatory cytokines, and hepatic genes altered at the transcription level. (E) Correlations of altered gut bacteria and gut metabolites with altered liver function indicators, inflammatory cytokines, and hepatic proteins. All of the results shown in the heatmap are significant at *P* < 0.05. The color key and circle size indicate the strength of the correlation (*r* value). Red indicates a positive correlation, and blue indicates a negative correlation.

In the gut, the positive correlations of *Firmicutes* (mainly *Lachnospiraceae* or *Ruminococcaceae*) members with carbohydrates and their metabolites accounted for nearly 70% of the total correlations among d-GalN-altered but LA14-enriched microbiota and metabolites (Fig. 5A). For example, *Coprococcus*, *Oscillospira*, and *Ruminococcus* were positively correlated with d-arabinose, d-glucose, and maltose. In addition, negative correlations were found for *Bacteroides* sp. dnLKV3 with butanoic acid, d-arabinose, d-glucose, and *N*-acetylglucosamine and for cyanobacteria with d-glucose and methyl galactoside.

In the liver, most of the d-GalN-altered but LA14-allevited transcription and/or expression of hepatic genes were intensively linked to liver function and inflammatory factors (Fig. 5B and C). First, the inflammatory factors AST, ALT, ALP, MIP-1α, MIP-3α, and MCP-1 were positively correlated with the transcription of genes related to cytokine secretion (*Lilrb4* and *Ackr3*) and cell carcinoma growth (*Rock2*, *Socs5*, *Serpine1*, *Plau*, *Plod2*, and *Col12a1*) and with proteins related to tumor-associated biomarkers (EMD, NAP1L1, STMN1, EZR, and STMN2), immune suppressors (KIF13B, TUBB5, UBE2D3, GBP7, ESAM, and CASP1), inducers of hepatic injury (KDSR and DEFA5), and lipid metabolic proteins (PON3, DDB1, RDH10, and LPL). TBA was positively correlated with the transcription of *Lpl* and *Col12a1* and proteins such as TMEM62 and STMN2. Second, the inflammatory factors AST, ALT, ALP, MIP-1α, MIP-3α, and MCP-1 were negatively correlated with the transcription of genes related to immunity regulation (*MstI*, *Ifi27*, and *Aspg*), cancer suppression (*Ftcd*, *Hpn*, *Pck1*, *Mat1a*, *Tdo2*, and *Clk1*), and lipid metabolism (*Eci1*, *Creb3l3*, *Fabp1*, and *Hsd17b13*) and with proteins related to immunosuppression (DNAJA1 and TIAL1), energy-related factors (LRPPRC, ABCB4, MT-ND5, ABCC6, and ARMC1), and proteins associated with dyslipidemia (LDLRAP1, ECHDC3, PTGES2, and PI4KA).

The gut microbiota and metabolites were also correlated with liver inflammation indicators and gene transcription or expression. An analysis of the gut microbiota revealed that *Coprococcus*, *Mediterraneibacter* sp. strain KCTC 15684, and Streptococcus sp. strain GDLAMI-SD2 were negatively correlated with the transcription or expression of genes related to cytokine secretion (*Lilrb4* and *Ackr3*) and cell carcinoma growth (*Rock2*, *Socs5*, *Serpine1*, *Plau*, *Plod2*, and *Col12a1*) but positively correlated with those related to cancer suppression (*Ftcd*, *Hpn*, *Pck1*, *Mat1a*, *Tdo2*, and *Clk1*) and lipid metabolism (*Eci1*, *Creb3l3*, *Fabp1*, and *Hsd17b13*). However, *Anaerostipes* sp. showed correlations opposite to those found for *Coprococcus*, *Mediterraneibacter* sp. KCTC 15684, and Streptococcus sp. strain GDLAMI-SD2. Furthermore, the d-GLAN-induced alterations in the gut microbiota with the exception of Lactobacillus faecis and *Anaerostipes* sp. were negatively correlated with inflammatory factors. The analysis of gut metabolites revealed that maltose, butanoic acid, methyl galactoside, *N*-acetylglucosamine, and d-arabinose were positively correlated with the transcription or expression of genes related to immunity regulation, lipid metabolism, and antioxidation and were negatively correlated with tumor-associated biomarkers.

### *L. acidophilus* LA14 can also alleviate acetaminophen-induced acute liver injury.

**FIG 6:**
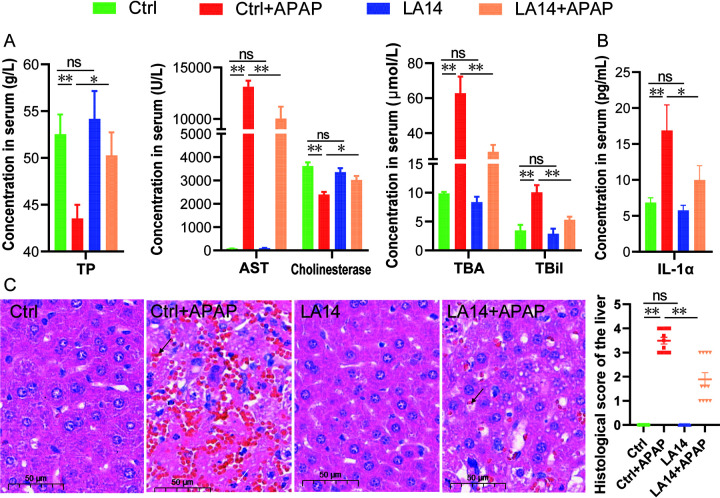
L. acidophilus LA14 also attenuated acetaminophen-induced liver injury. (A) Liver function of each group. (B) Hepatic inflammatory cytokine IL-1α in each group. (C) Representative images of hepatic HE staining and histological scores of the livers. Scale bar, 50 μm. The black arrow points to hemorrhage, and the white arrow indicates nuclear shrinkage. The data are shown as means ± the SEM (*, *P* < 0.05; **, *P* < 0.01; ns, the difference is not significant).

The effect of LA14 on acute liver injury was further verified using a mouse model of acetaminophen hepatotoxicity. The analysis of hepatic function showed that pretreatment with LA14 significantly reduced the acetaminophen-induced decrease in the serum level of total protein (TB), increase of AST, cholinesterase, TBAs, and total bilirubin (TBil) (Fig. 6A). Furthermore, a histological examination showed that LA14 pretreatment significantly reduced the symptoms of liver hemorrhage, hepatic cellular nuclear shrinkage and inflammatory cell infiltration caused by acetaminophen and reduced the overall pathological score (Fig. 6C). In addition, LA14 reduced the elevation of IL-1α in the serum of acetaminophen-treated mice (Fig. 6B). These results suggested that LA14 might exert a good preventive effect on liver injury induced by multiple chemicals.

## DISCUSSION

Acute liver injury is a severe hepatic disease caused by many factors, such as drugs, viruses, alcohol, and autoimmunity (9). This disease has emerged as a public health issue around the world and can present as a life-threatening disorder (1). L. acidophilus LA14 is a widely used probiotic worldwide. In the present study, pretreatment with LA14 alleviated d-GalN-induced liver injury in rats, as indicated by reductions in the elevations of serum ALT, AST, ALP, and TBAs; decreases in inflammation and necrosis in the liver and terminal ileum; and the minimization of dysbiosis of the gut microbiota and metabolome after pretreatment. The underlying mechanism of LA14 might include alleviation of the d-GalN-induced downregulation of antioxidation-related pathways and upregulation of infection- and inflammation-related pathways.

Our results showed that L. acidophilus LA14 reduced d-GalN-induced liver injury. First, LA14 alleviated the d-GalN-induced elevations in ALT, AST, ALP, and TBAs. Among these factors, ALT is mainly produced and exists in hepatocytes, and the levels of ALT are nearly 100-fold higher in the liver than in serum. AST is mainly produced by the liver, although other organs, such as the heart, kidneys, brain, and muscles, also produce small amounts of AST. ALP is predominantly present in the liver and bones and is an indicator of the state of absorption and transport in the cell membrane (14). Bile acids are synthesized from cholesterol in the liver and released into the intestine (16). Therefore, the alleviation of the increases in these compounds indicates decreased damage to hepatic tissue, which is in line with our HE staining observations that LA14 alleviates hepatic histological abnormalities, such as hepatocyte loss and lipid vacuole accumulation. Second, pretreatment with LA14 alleviated the d-GalN-induced increase in serum chemokines, including MIP-1α, MIP-3α, and MCP-1, which recruit and activate monocytes/macrophages to the injured tissue area (10). This finding is consistent with our results that LA14 alleviated the infiltration of inflammatory cells in liver tissue and reflects the protective effects of LA14 on liver injury.

Our results showed that LA14 alleviated d-GalN-induced liver injury and simultaneously restored some d-GalN-induced gut microbiota dysbiosis. Several d-GalN-altered but LA14-alleviated gut bacterial taxa are reportedly significantly altered and associated with liver diseases in patients. For example, harmful cyanobacterial blooms are a potential risk factor for nonalcoholic liver disease, as revealed in a country-level ecological study in the United States (17); *Bacteroides* is significantly enriched in patients with nonalcoholic steatohepatitis and severe fibrosis, whereas *Ruminococcus* is significantly enriched in patients with severe fibrosis (18); depletion of *Oscillospira* and *Coprococcus* has been observed in patients with persistent nonalcoholic fatty liver disease (19); and *Lachnospira* is positively correlated with lipid metabolism, and its depletion is associated with lipid metabolic disorders (20). However, this study still observed some alterations in bacteria, such as *Acutalibacter* and Lactobacillus faecis, whose roles in liver diseases remain undetermined. Notably, LA14 administered by gavage was not detected in rat feces, and this failure was mainly due to the limited reading length of second-generation sequencing, which results in accurate classification usually to the genus level and very few species (21). In addition, the administration of LA14 did not enrich fecal *Lactobacillus* in either the LA14 group or the LA14+GalN group, which suggested that LA14 contributes little to the relative abundance of fecal *Lactobacillus* detected in this work.

Our results showed that LA14 attenuated d-GalN-induced fecal metabolome disorder. Some metabolites involved in this process are reportedly important to liver diseases. For example, butanoic acid reportedly not only attenuates ConA-induced acute liver injury in mice by inhibiting the expression and release of HMGB (22) but also protects against inflammation by reducing cytokine production (23). Arabinose, when administered in combination with the same amount of mannose, attenuates liver injury induced by CCL4 through an anti-inflammatory mechanism (24). d-Glucose is a carbon unit for hepatic *de novo* lipogenesis and thereby contributes to nonalcoholic fatty liver disease and provides energy (25). Maltose, similar to d-glucose, is generated in the gut during starch digestion as a carbon transfer molecule and energy reserve (26), and its role in liver diseases is not fully understood. *N-*Acetyl-d-glucosamine is best known as a component of cell wall peptidoglycans in bacteria, cell wall chitin in fungi and parasites, the exoskeletons of arthropods, and the extracellular matrix of animal cells. This compound is primarily used to enhance the immune system, to reduce inflammation and to inhibit the excessive growth of cancer or fiber cells (27). Moreover, *N-*acetyl-d-glucosamine reportedly antagonizes liver injury and the oxidative damaging effect of both paracetamol and phenacetin (28). Glycerol monostearate, which is known as the best lipid conjugate, is often used as a carrier of lipids (29). Therefore, this evidence suggests that LA14 might provide resistance to d-GalN-induced liver injury through metabolic regulation.

A transcriptome analysis of the liver and terminal ileum revealed that the regulation of tumor- and antioxidation-related pathways might be involved in the mechanism through which LA14 alleviates liver injury. First, 35 liver genes and one gut gene might be involved in the alleviation of liver injury by LA14. The products of these liver genes are important for mitigating inflammation, preventing hepatocellular carcinoma and regulating lipid metabolism. For example, ACKR3, which was previously known as C-X-C chemokine receptor type 7 (CXCR7), is a main regulator of physiological processes at steady state and during inflammation (30). SOCS5 inhibition induces autophagy to impair metastasis in hepatocellular carcinoma cells via the PI3K/Akt/mTOR pathway (31). cAMP responsive element-binding protein 3-like 3 (CREB3L3) is a basic, liver-specific leucine zipper transcription factor involved in fatty acid oxidation and ketogenesis in synergy (32). Notably, the regulation of these genes was highly consistent with the declines in serum ALT, AST, ALP, TBA, MCP-1, MIP-3α, and MIP-1α observed after pretreatment with LA14. Second, a KEGG pathway analysis of our transcriptome results further revealed that LA14 might alleviate d-GalN-induced liver injury by regulating pathways that fight against inflammation, lipid metabolism disorders and tumorigenesis in the liver. ECM-receptor interaction pathway and proteoglycans in the cancer pathway are upregulated by d-GalN, which this upregulation is alleviated by pretreatment with L. acidophilus LA14; these pathways are involved in tumorigenesis (33, 34). In particular, proteoglycans in cancer are often used as biomarkers and therapeutic targets in hepatocellular carcinoma (34). In addition to tumorigenesis, ascorbate and aldarate metabolism is an alternative pathway to enhance the antioxidant capacity in response to stress (35), and the cysteine and methionine metabolism pathway is involved in oxidative stress, protein damage, and repair.

The proteomic analysis of the liver showed that infectious and inflammatory pathways might also be involved in the mechanism through which liver injury is attenuated by LA14. For example, LA14 alleviated the d-GalN-induced upregulation of complement and coagulation cascades, the majority of which were mainly biosynthesized and secreted by the liver, mainly hepatocytes. It has been reported that activation of the coagulation pathway was accompanied by activation of the complement cascade during episodes of tissue injury and inflammation (36). Similar to many previous studies (37, 38), our results showed poor correlations between the transcriptome and proteome results. One reason for this finding is that transcription and translation are two coherent but relatively independent processes, and significant differences in half-life, synthesis rate, and quantity are found between proteins and mRNAs (38). Another reason might be that translation does not simply copy the information of mRNA but rearranges the information, which results in the transcript abundance not being equal to the protein abundance (38). However, genes that significantly contributed to the LA14-mediated alleviation of liver injury selected from the transcriptome and proteome were both strongly correlated with indicators of liver injury and cytokines.

Our results showed that eight liver genes were significantly involved in the alleviation of liver injury by LA14 at both the transcriptional and expression levels. ROCK2 (Rho-associated coiled-coil containing kinases 2) is a member of the serine/threonine protein kinase family, which mediates effects downstream of the small GTP-binding protein RHOA. ROCK2 promotes hepatocellular carcinoma via the ubiquitination of CDC25A (39). FBLIM1 (filamin-binding LIM protein 1) has previously been described to promote hepatocellular cancer progression by sponging miR-346 (40). COL12A1 (collagen type XII α1 chain) is a member of the fibril-associated collagen family and has received increasing attention due to its essential roles in human cancer because its overexpression has been identified in several different cancer types (41). LA14 alleviated the d-GalN-induced upregulation of ROCK2, FBLIM1, and COL12A1, which suggested that LA14 might contribute to the prevention of hepatocellular cancer during acute liver injury. LPL (lipoprotein lipase) is the key enzyme of lipid metabolism and is involved in the hydrolysis of triacylglycerol-rich lipoproteins (TRLs) to release fatty acids (42). Liver LPL overexpression or deficiency has recently revealed glucose and lipid metabolic disorders in mice (43). SOD1 (superoxide dismutase 1) is commonly known for its reactive oxygen species (ROS) scavenging activity. Therefore, these genes and their related pathways might play important roles in the alleviation of d-GalN-induced liver injury by LA14.

As a probiotic, L. acidophilus LA14 is more often used by healthy people for health care and disease prevention. Therefore, the effect of LA14 on healthy rats was another focus of this study. First, the oral administration of L. acidophilus LA14 did not alter biochemical indicators of hepatic function, cytokines or histology of the liver and ileum. These results suggest that LA14 exerts no harmful effect on the liver and gut. Second, the oral administration of LA14 enriched several beneficial bacteria, such as *Bifidobacterium*, and depleted several potentially opportunistic pathogens, such as *Oscillospira* and *Lachnospira* (22–24), which suggested that LA14 might promote gut health. Third, the oral administration of LA14 altered the distributions of several metabolites, which appears to be beneficial for health. For instance, a lower content of 5-hydroxyhexanoic acid reportedly predicts early renal functional decline in patients with type 2 diabetes accompanied by microalbuminuria (42), and the increase in 5-hydroxyhexanoic acid induced by LA14 might aid resistance to type 2 diabetes.

The ability of LA14 to alleviate liver injury was further confirmed with an acetaminophen-induced mouse model. Acetaminophen (APAP) has been widely used as an analgesic and anti-pyretic agent, which overdose is the primary cause of acute liver failure in several countries (44). Mice are the preferred animal for studies of APAP overdose, because they have the same mechanisms of toxicity, including glutathione depletion, protein binding, mitochondrial damage, oxidative stress, DNA fragmentation, and Jnk activation (44). LA14 was also observed to alleviate liver injury in the mouse by alleviating APAP-induced reduction of TB and elevation of AST, cholinesterase, TBAs, TBil, and IL-1α in the serum, as well as a decrease in hemorrhage and inflammation in the liver after pretreatment.

**FIG 7:**
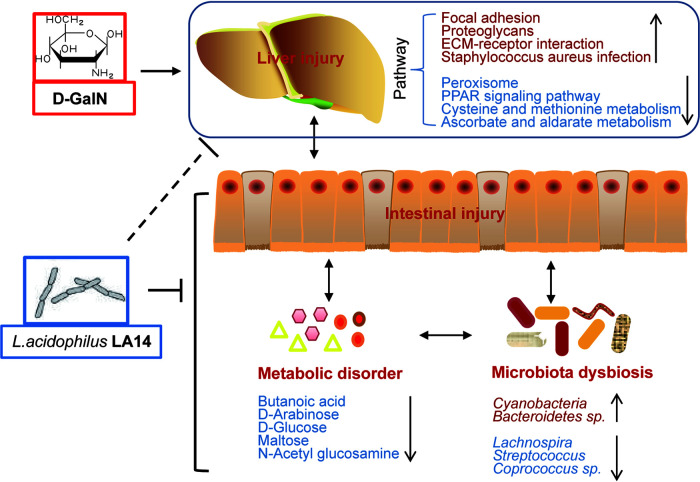
General view of the beneficial effects of L. acidophilus LA14 on acute liver injury.

In summary, we demonstrate here that L. acidophilus LA14 alleviates d-GalN-induced liver injury in rats. The underlying mechanism of this effect of LA14 includes alleviation of the d-GalN-induced downregulation of antioxidation-related pathways, reduction of the d-GalN-induced upregulation of infection- and inflammation-related pathways, and restoration of the gut microbiota and metabolome homeostasis (Fig. 7). These results, together with the good performance of this treatment in healthy rats, might facilitate the application of L. acidophilus LA14 in the prevention and treatment of liver diseases.

## MATERIALS AND METHODS

### Strains and culture conditions.

Lactobacillus acidophilus LA14 was supplied by DuPont. The strain was cultured anaerobically in MRS agar (Thermo Fisher, Shanghai, China) for 48 h at 37°C, identified by 16S ribosomal DNA sequencing, and stored at −80°C for further use. Before the experiment, all the strains were cultured in MRS broth (Thermo Fisher) at 37°C for 24 h. Cells were collected by centrifugation at 8,000 × *g* and 4°C for 10 min, resuspended in phosphate buffer to a final concentration of 3 × 10^9^ CFU/ml, and used for further animal experiments (9).

### Animals and experimental design.

Male Sprague-Dawley rats with an initial weight of 250 to 300 g were assigned to four groups with randomized blocks of seven to nine replications. The rats in the LA14 and LA14+GalN groups were orally administered the LA14 suspension (3 × 10^9^ CFU/ml) for 7 days, whereas those in the Ctrl and Ctrl+GalN groups were orally administered PBS for 7 days. On day 8, the rats in the LA14+GalN and Ctrl+GalN groups received an intraperitoneal injection of d-GalN (1.1 g/kg body weight; G0500, Sigma, St. Louis, MO) to induce acute liver injury, whereas those in the Ctrl and LA14 groups were intraperitoneally injected with saline.

At 24 h after d-GalN injection, fecal samples were collected from all the animals, and the animals were then aseptically anaesthetized with 30 mg/kg pentobarbital sodium (Sigma) and sacrificed. Blood was collected from the inferior vena cava and centrifuged at 3,000 × *g* for 10 min to obtain serum samples for the assessment of biochemical indicators of hepatic function and cytokine analysis. The samples of the liver (approximately 8 mm × 8 mm) and terminal ileum (approximately 10 mm in length) for histological evaluation were immediately fixed in 10% paraformaldehyde. The other liver and terminal ileum samples were stored in liquid nitrogen for further study.

All experimental procedures were performed in accordance with the 2011 National Institutes of Health *Guide for the Care and Use of Laboratory Animals*. The study protocol was approved by the Animal Care Committee of Zhejiang University, China.

### Biochemical indicators of hepatic function.

The serum levels of glycylproline dipeptidyl aminopeptidase, ALT, AST, ALP, cholinesterase, total bilirubin, total bile acids, direct bilirubin, total protein, and albumin were tested using a Hitachi 7600-210 automatic analyzer (Tokyo, Japan) (43).

### Cytokine analysis.

A Bio-Plex Pro Rat or Mouse Cytokine 23-Plex Panel magnetic bead suspension array (Bio-Rad, Hercules, CA) was used for the analysis of serum cytokines based on the manufacturer’s instructions. The kits included vascular endothelial growth factor, IFN-γ, TNF-α, G-CSF, granulocyte-macrophage colony-stimulating factor, GRO/KC, RANTES, macrophage colony-stimulating factor, MIP-1α, MIP-3α, MCP-1, IL-1α, IL-1β, IL-2, IL-4, IL-5, IL-6, IL-7, IL-10, IL-12, IL-13, IL-17, and IL-18.

### Histological examination.

The left lobe of the liver and terminal ileum was fixed with 10% formaldehyde solution for 24 h and embedded in paraffin. The tissue was cut into 2-μm-long sections and stained with HE. The liver tissue damage was assessed using a histological score obtained semiquantitatively from the histological activity index (44), which consists of two categories. One category evaluates intralobular degeneration and focal necrosis and is given a score of 0 to 1 or 3 to 4, and the other assesses the degree of portal inflammation representing acute liver damage and is given a score of 0 to 1 or 3 to 4. Intestinal mucosal lesions were evaluated as previously described (45). A score of 0 indicates a normal mucosa, whereas a score 1 signifies the development of subepithelial Gruenhagen’s space at the tip of the villus, and a score of 2 indicates greater extension of Gruenhagen’s space. Tissue damage was evaluated using at least three slides from each specimen.

### Gut microbiota analysis.

DNA was extracted from 0.2 g of stool using the QIAamp DNA stool minikit (Qiagen, Valencia, CA). The V3-V4 regions of the bacterial 16S rRNA gene sequences were amplified using the PCR primer pairs 343F (5′-TACGGRAGGCAGCAG-3′) and 798R (5′-AGGGTATCTAATCCT-3′). PCR was performed in a 25-μl PCR system containing 50 ng of template DNA, 25 pmol of each primer, and 12.5 μl of Phusion Hot Start Flex 2× Master Mix (New England Biolabs, Beijing, China), and the program consisted of initial denaturation at 98°C for 30 s, 32 cycles of amplification (denaturation at 98°C for 10 s, annealing at 54°C for 30 s, and extension at 72°C for 45 s), and a final extension at 72°C for 10 min. The amplicon was purified with AMPure XP beads (Agencourt, Beckman Coulter, USA) and sequenced using Illumina MiSeq platforms (Illumina, Inc., San Diego, CA).

FLASH (version 1.2.11) was used to assemble paired-end reads after the trimming process (45). The following quality control criteria were used: (i) an exact match to at least one end of barcodes and primers; (ii) no undetermined bases in the tags; and (iii) at most three mismatches in the overlap region. OTUs were clustered with a 97% similarity cutoff using Usearch 11 (46). The longest sequence in each OTU was selected as the representative sequence for genus and species identification using the Ribosomal Database Project (RDP) database v.11.3 (47) and NT-16S (48), respectively. For each observed species, PCoA of unweighted UniFrac beta diversity was performed using QIIME software (version 1.8.0) to investigate the richness and diversity of each gut microbiome (49).

### Metabolomic analysis.

The metabolomic sample was prepared from 0.2 g of feces as described previously (50, 51). Metabolites were assayed by gas chromatography-mass spectrometry (GC-MS) using an Agilent 7890A GC system coupled to an Agilent 5975C inert mass selective detector system (Agilent Technologies, Santa Clara, CA). One microliter of prepared sample was injected into an Agilent J&W Hp-5 MS column in the pulsed splitless mode. The injection and interface temperatures were set to 250°C. The initial GC oven temperature was first maintained at 60°C for 5 min, then increased to 70°C for 2 min, further increased to 200°C at 10°C/min, maintained for 10 min, increased at a rate of 4°C/min to 300°C, and maintained at 300°C for 2 min. The carrier gas was helium at a flow rate of 1.0 ml/min. The solvent delay was set to 5 min 25 s. The measurements were made with electron impact (EI) ionization (70 eV) in the full-scan mode (50 *m/z* to 600 *m/z*).

Metabolites were identified using the NIST databases (https://www.nist.gov/) (score > 80). Orthogonal partial least-squares-discriminant analysis (OPLS-DA) and variable importance in the projection (VIP) calculations were performed using SIMCA software (version 14.1; Sartorius Stedim Biotech, Umeå, Sweden).

### Comparative transcriptome analysis.

Total RNA of the liver and terminal ileum was extracted using the TRIzol reagent (Invitrogen, Carlsbad, CA) and purified using an RNA 1000 Nano LabChip kit (Agilent, Santa Clara, CA). The cDNA library was constructed using the mRNA-Seq sample preparation kit (Illumina, San Diego, CA). The cDNA library was then sequenced with an Illumina HiSeq 4000 (Illumina), and 150-bp paired-end reads were generated.

The raw sequence data were first processed using in-house Perl scripts to remove reads containing adapters, reads containing poly-N and low-quality reads. The clean reads were aligned to the UCSC (http://genome.ucsc.edu/) rat reference genome using HISAT2 (version 2.0.5), assembled using StringTie (version 1.3.3b), and merged to reconstruct a final transcriptome using Perl scripts (52). StringTie (version 1.3.3b) and featureCounts (version 1.5.0-p3) were then utilized to determine the expression levels of mRNAs by calculating fragments per kilobase of exon model per million mapped reads (FPKM) (53, 54).

A differential transcription analysis of two groups was performed using the DESeq2 R package (version 1.20.0). The resulting *P* values were adjusted using Benjamini and Hochberg’s approach for controlling the false discovery rate. Genes with a *P*_adj_ of <0.05 identified by DESeq2 were assigned as differentially expressed transcripts.

### Proteomics analysis.

The liver samples stored in liquid nitrogen were used for proteomic analysis. Total proteins were extracted from 50 mg of samples as described previously (55). TMT labeling of peptides was performed using TMT Mass Tagging Kits and Reagents (Thermo, Rockford, IL) according to the manufacturer’s instructions. Peptide fractionation and LC-MS/MS for proteomics analyses were then also performed according to a previous study (55).

The resulting spectra were searched in the NCBI nr (https://www.ncbi.nlm.nih.gov/), UniProt (http://www.uniprot.org/) and BioGRID (https://thebiogrid.org/) databases using the search engine Proteome Discoverer 2.4 (PD 2.4; Thermo). The search parameters were set as follows: a mass tolerance for precursor ions of 10 ppm and a mass tolerance for product ions of 0.02 Da.

The differential expression analysis of two groups was performed using the DESeq2 R package (version 1.20.0). The resulting *P* values were adjusted using Benjamini and Hochberg’s approach for controlling the false discovery rate. Genes with a *P* value of <0.05 identified by DESeq2 were considered differentially expressed. Genes belonging to different groups in the Venn diagram were plotted using the VennDiagram R package (version 1.6.20), and the enrichment of differentially expressed genes in KEGG pathways was calculated and plotted using the clusterProfiler (version 3.5.1) and ggplot2 (version 3.1.1) packages.

### Methods for verifying the hepatoprotective effect of *L. acidophilus* LA14.

To verify the hepatoprotective effect of LA14, a mouse model of acetaminophen-induced acute liver injury was established (56). Male C57BL/6J mice with an initial weight of 20 to 22 g were assigned to four groups with randomized blocks of 10 to 12 replications. The mice in the LA14 and LA14+APAP groups were orally administered 0.2 ml of the LA14 suspension (3 × 10^9^ CFU/ml) for 7 days, whereas those in the Ctrl and Ctrl+APAP groups were orally administered PBS for 7 days. On day 8, the mice in the LA14+APAP and Ctrl+APAP groups were orally administered acetaminophen (APAP; 300 mg/kg body weight in 0.5% sodium carboxy methylcellulose) to induce acute liver injury, whereas those in the Ctrl and LA14 groups were gavaged with the same dose of 0.5% sodium carboxy methylcellulose. At 24 h after acetaminophen administration, all the animals were aseptically anaesthetized with 30 mg/kg pentobarbital sodium (Sigma) and sacrificed. Blood was collected from the inferior vena cava and centrifuged at 3,000 × *g* for 10 min to collect serum samples for the assessment of biochemical indicators of hepatic function and cytokines. The samples of the liver (approximately 5 mm × 5 mm) for histological evaluation were immediately fixed in 10% paraformaldehyde after their isolation.

### Statistical analysis.

The obtained data were analyzed using SPSS 22.0 software (SPSS Inc., Chicago, IL). The data were first tested using the Kolmogorov-Smirnov test to determine the normality of their distribution. The Mann-Whitney U test (for nonnormal distributions) or Student *t* test (for normal distributions) was used to assess the differences between two groups. Spearman’s rank correlation analysis was conducted using the corrplot package of R. Unless otherwise specified, the data are shown as means ± the standard errors of the mean (SEM).
