# Supplementary figures and images for: Lactobacillus acidophilus LA14 Alleviates Liver Injury
Source: mSystems. 2021 Jun 15;6(3):e00384-21. doi: 10.1128/mSystems.00384-21 (PMC8269229; doi:10.1128/mSystems.00384-21)

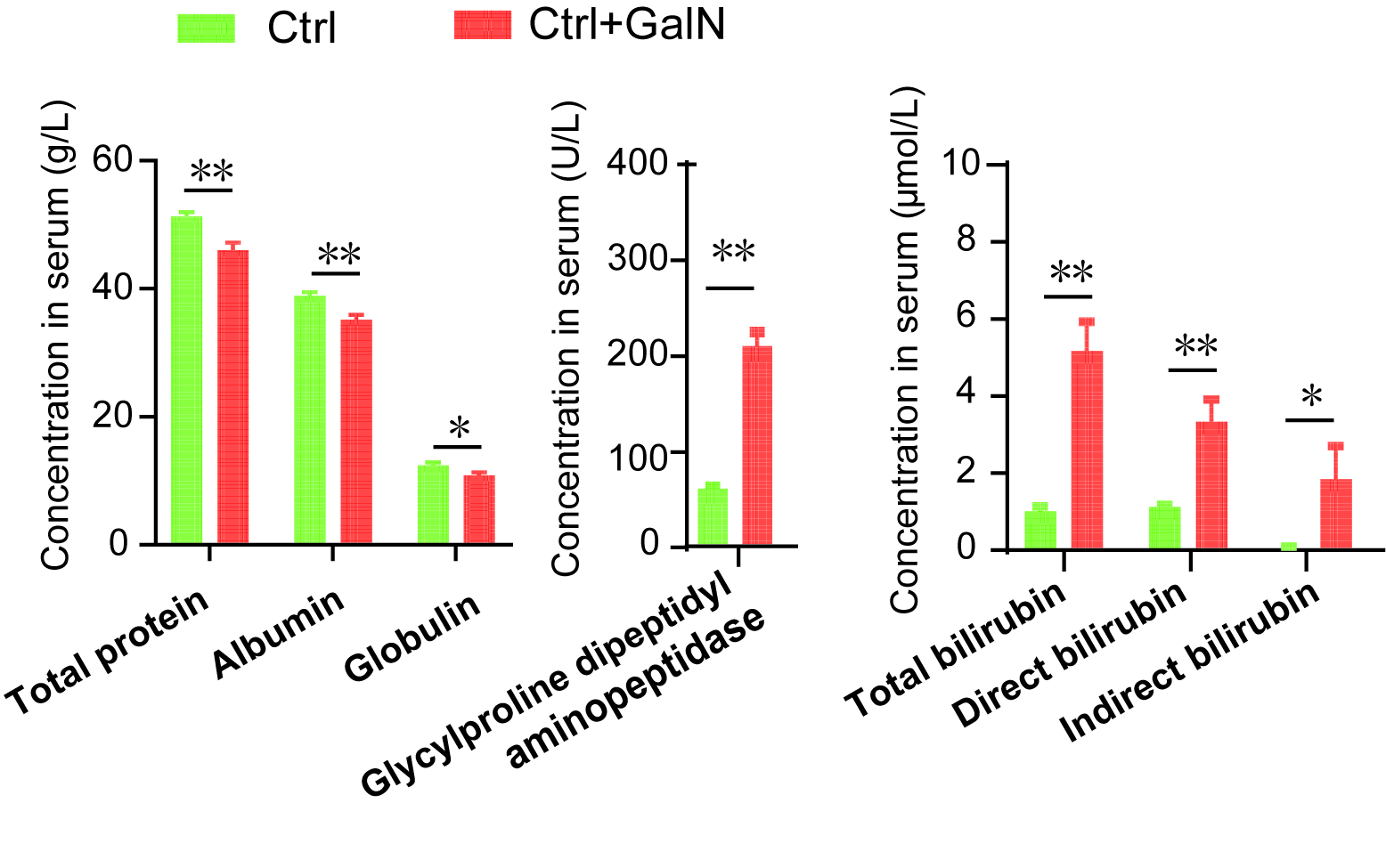

Supplement: FIG S1 [file msystems.00384-21-sf001.tif]

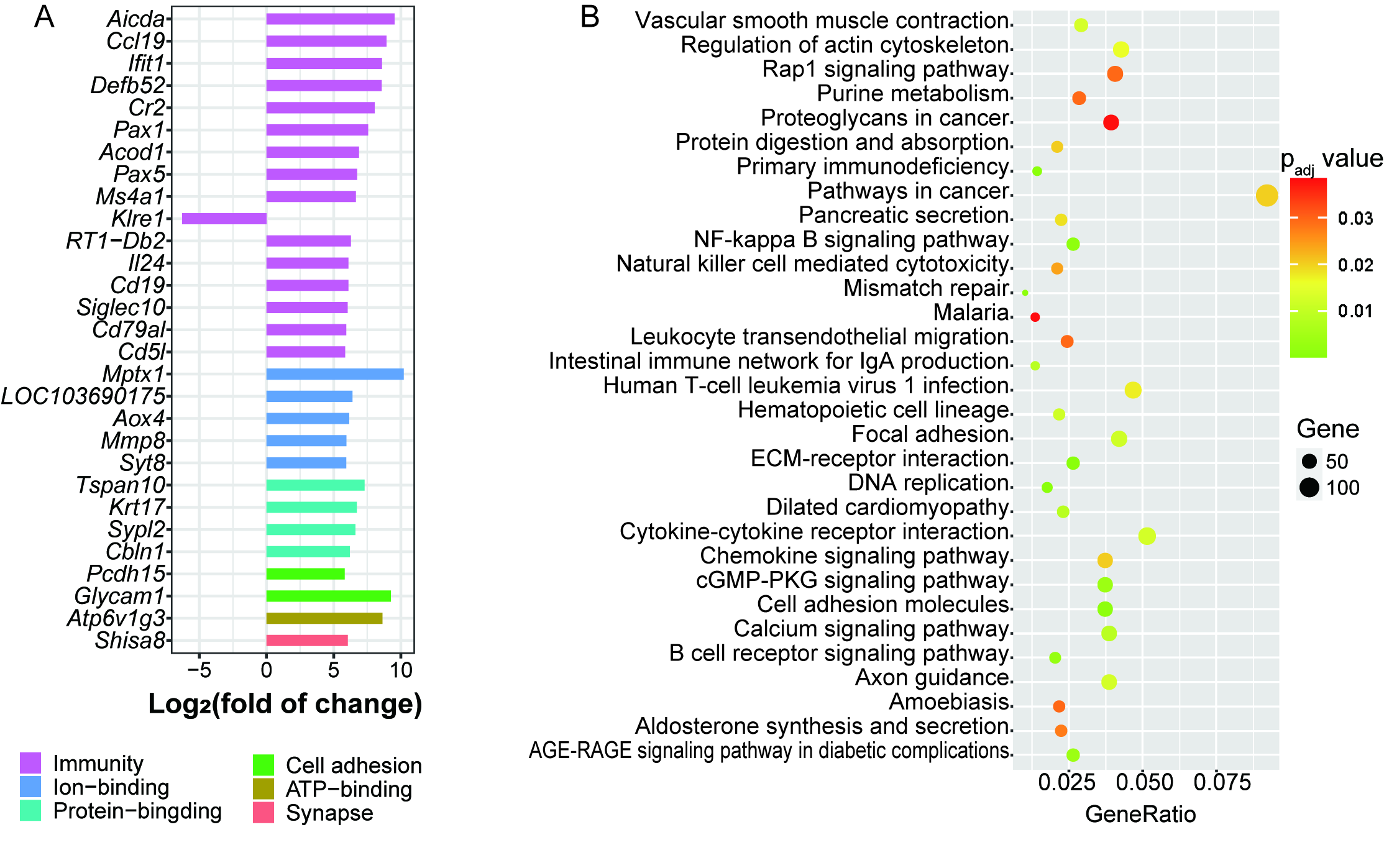

Supplement: FIG S2 [file msystems.00384-21-sf002.tif]
